# Hospitalizations among older adults: results from ELSI-Brazil

**DOI:** 10.11606/S1518-8787.2018052000639

**Published:** 2018-10-25

**Authors:** Alexandre Moreira de Melo-Silva, Juliana Vaz de Melo Mambrini, Paulo Roberto Borges de Souza, Fabíola Bof de Andrade, Maria Fernanda Lima-Costa

**Affiliations:** I Fundação Oswaldo Cruz. Instituto René Rachou. Programa de Pós-Graduação em Saúde Coletiva. Belo Horizonte, MG, Brasil; II Fundação Oswaldo Cruz. Instituto René Rachou. Núcleo de Estudos em Saúde Pública e Envelhecimento. Belo Horizonte, MG, Brasil; III Fundação Oswaldo Cruz. Instituto de Comunicação e Informação Científica e Tecnológica em Saúde. Rio de Janeiro, RJ, Brasil

**Keywords:** Aged, Hospitalization, Chronic Disease, Cardiovascular Diseases, epidemiology, Idoso, Hospitalização, Doença Crônica, Doenças cardiovasculares, epidemiologia

## Abstract

**OBJECTIVE:**

To examine the factors associated with hospital use and their frequency in a nationally representative sample of the Brazilian population aged 50 years or older.

**METHODS:**

Data from the baseline of the Brazilian Longitudinal Study of Aging (ELSI-Brazil), conducted in 2015-2016, were used. Predisposing, enabling and need factors for the use of health services were considered. The analyzes were based on the Hurdle regression model and on estimates of population attributable risks.

**RESULTS:**

Among 9,389 participants, 10.2% had been hospitalized in the previous 12 months. After adjusting for potential confounding variables, statistically significant associations (p < 0.05) were observed for need factors (previous medical diagnosis for chronic diseases and limitation to perform basic activities of daily living) and for enabling factors (living in a rural area and in the North and Midwest regions of the country). The analysis of population attributable risks (PAR) showed a hierarchy of the need factors for the occurrence of hospitalizations, with higher contributions by stroke (PAR = 10.7%) and cardiovascular disease (PAR = 10.0%), followed by cancer (PAR = 8.9%), difficulty to perform basic activities of daily living (PAR = 6.8%), depression (PAR = 5.5%), diabetes (PAR = 4.4% ) and hypertension (PAR = 2.2%).

**CONCLUSIONS:**

Four of the major diseases associated with hospitalizations (stroke, cardiovascular disease, diabetes and hypertension) are part of the Brazilian list of primary care-sensitive hospitalizations. These results show that there is a window of opportunity to reduce unnecessary hospitalizations among older Brazilian adults through effective primary care actions.

## RESUMO

**OBJETIVO:**

Examinar os fatores associados à ocorrência e à frequência de hospitalizações em amostra nacional representativa da população brasileira com 50 anos ou mais.

**MÉTODOS:**

Foram utilizados dados da linha de base do Estudo Longitudinal da Saúde dos Idosos Brasileiros (ELSI-Brasil), conduzido em 2015–2016. Considerou-se fatores predisponentes, facilitadores e de necessidade para o uso de serviços de saúde. As análises foram baseadas no modelo de regressão *Hurdle* e em estimativas de riscos atribuíveis populacionais.

**RESULTADOS:**

Entre 9.389 participantes, 10,2% foram hospitalizados nos 12 meses precedentes. Após ajustes por potenciais variáveis de confusão, associações estatisticamente significantes (p < 0,05) foram observadas para fatores de necessidade (história de diagnóstico médico para doenças crônicas e limitação para realizar atividades básicas de vida diária) e para fatores facilitadores (residência em zona rural e nas regiões Norte e Centro-Oeste do país). A análise dos riscos atribuíveis populacionais (RAP) mostrou uma hierarquização dos fatores de necessidade para a ocorrência de hospitalizações, com maiores contribuições do acidente vascular cerebral (RAP = 10,7%) e da doença cardiovascular (RAP = 10,0%), seguidos do câncer (RAP = 8,9%), da limitação para realizar atividades básicas da vida diária (RAP = 6,8%), da depressão (RAP = 5,5%), do diabetes (RAP = 4,4%) e da hipertensão (RAP = 2,2%).

**CONCLUSÕES:**

Quatro entre as principais doenças associadas às hospitalizações (acidente vascular cerebral, doença cardiovascular, diabetes e hipertensão) fazem parte da lista brasileira de internações sensíveis à atenção primária. Esses resultados mostram que existe uma janela de oportunidades para a redução de hospitalizações desnecessárias entre adultos brasileiros mais velhos por meio de ações efetivas da atenção primária.

## INTRODUCTION

The rapid aging of the population poses numerous challenges to Brazilian society and to other middle- and low-income countries^1^. Health systems, in particular, have adapted to the growing demand for medical consultations, propaedeutics, medical procedures and hospitalizations, which entail growing costs^2^. Hospital services are complex and costly and, proportionally, used more by the aged^2^. A better understanding of the use of these services by this population can contribute to planning of healthcare, as well as to prevent avoidable hospitalizations and reduce inequities.

In 2016, the Brazilian Public Health System (*Sistema Único de Saúde* - SUS) performed more than 11 million hospital admissions at a cost of approximately 14 billion reais (i.e., USD 4 billion). Of these hospitalizations, 36% were for people aged 50 or older, which consumed about 48.5% of the above-mentioned resources^2^. The prospect is that the number of hospitalizations will increase in coming years as a result of population aging. The magnitude of health spending will be strongly influenced by the tension between healthy aging and those with the greatest burden of illness and dependence. These data reinforce the need for the health system to remove barriers to access, promote effective coordination of care, and focus on health promotion and the prevention of morbidity and disability^3^.

The theoretical construct of the use of health services can be systematized from three axes: the characteristics of the health system, the scientific transformations, and the social norms that intermediate the medical conduct and the individual determinants of use^4^. The Andersen and Newman behavioral model^4^ was constructed from the individual determinants of the use of these services. This model is based on predisposing factors (such as gender and age), enabling factors (such as a socioeconomic condition) and needs (such as health conditions), and has been widely used in different countries, including Brazil^5^ ^,^ ^6^. Such health conditions include morbidities^7^ ^-^ ^11^, fragility^12^ and functionality^6^ ^,^ ^11^, and are particularly relevant in the occurrence of hospitalizations^7^ ^,^ ^9^ ^,^ ^11^ ^,^ ^13^. We are not aware of studies based on a national sample that examines the contribution of different need factors to the occurrence of hospitalizations among Brazilian older adults.

The present study aimed to identify factors associated with hospitalizations in a nationally representative sample of the Brazilian population aged 50 or older, with emphasis on the contribution of different need factors to the outcome.

## METHODS

Data from the baseline of the Brazilian Longitudinal Study of Aging (ELSI-Brazil), collected between 2015 and 2016, were used. The ELSI-Brazil sample was designed to represent the non-institutionalized Brazilian population aged 50 years or older. It is a complex sample, based on different selection stages, which consider the municipality, the census tract and the household. The sample size was estimated at 10,000 subjects. The baseline survey included 9,412 participants, living in 70 municipalities in the five major Brazilian regions. More details on the research can be found on the ELSI-Brazil homepage^a^ and in another publication^14^.

The outcome variable of this study was hospital use in previous 12 months, as measured by the answers to the following questions: “In the last 12 months, have you been hospitalized for 24 hours or more?” and, “In the last 12 months, how many times have you been hospitalized?”.

The selection of independent variables for the present analysis was based on the Andersen and Newman theoretical framework^4^. The predisposing factors were age and gender. Among the enabling factors, the place of residence (urban area and rural area), residence in large Brazilian geographic regions (North, Northeast, Midwest, Southeast and South), education (stratified into none, between one and four years, between five and eight years, and more than nine years), living arrangements (living alone, with one person or with two or more persons), private health plan coverage (yes, no), and an asset score, used as an indicator of the socioeconomic conditions of the family. This score was calculated by analyzing the main components, based on existing equipment at home (household appliances and automobiles) and the presence of domestic workers. The values of this score range from -∞ to +∞. Higher values represent better conditions. For this analysis, the asset score was divided into quartiles.

Lastly, among the need factors, we considered ability to perform basic activities of daily living (BADL) and the history of medical diagnosis for different chronic diseases, including cardiovascular disease (angina, heart failure or myocardial infarction), hypertension, diabetes mellitus, stroke, depression and cancer. The limitation to perform BADL was defined by reporting any difficulty to perform one or more of the following activities: walking across a room, getting in and out of bed, dressing, bathing, using the toilet and eating.

The results were described in percentages and the respective 95% confidence intervals (95%CI). In the unadjusted analyzes, Pearson’s chi-squared test, corrected for weighted data^15^, was used to examine the statistical significance of the differences between proportions.

Multivariate analyzes of the factors associated with hospitalizations and their frequencies were performed using the Hurdle regression model^16^. This model is composed of two functionally independent parts: the first considers the outcome variable as binary; the second uses a truncated model and considers only the positive counts^16^ ^,^ ^17^. The first step is modeled by logistic regression and produces estimates of odds ratio (OR); the second, by Poisson regression, and produces estimates of relative risks (RR). All predisposing, enabling and need variables were simultaneously included in the multivariate models, after verifying that they were not collinear (variance inflation factor < 5).

In addition, we estimated the population attributable risks (PAR) associated with the different need factors for hospitalizations. For this purpose, the regpar command of the Stata^18^ software was used. These estimates were obtained through a completely adjusted model, that is, simultaneously adjusted by the predisposing, enabling and need factors.

For all analyzes, weights of individuals and sample parameters were considered by procedures for complex samples of Stata software, version 13.0.

ELSI-Brazil respects the parameters contained in the Declaration of Helsinki and was approved by the Research Ethics Committee of the Fundação Oswaldo Cruz, Minas Gerais (CAAE 34649814.3.0000.5091).

## RESULTS

Of the 9,412 participants in the ELSI-Brazil baseline survey, 9,389 had complete information for all variables and were included in the present analysis. Among them, the average age was 63.0 years and 54.0% were women. One or more hospitalizations in the previous 12 months was reported by 10.2% of the participants. The corresponding values were 8.7%, 11.2%, 11.6%, 13.6% in the age groups of 50-59, 60-69, 70-79 and 80 years or older, respectively. For those aged 60 years or older, 11.6% had at least one hospitalization during the above mentioned. The most frequent chronic disease was hypertension (52.2%), followed by depression (18.6%), diabetes (15.8%), cardiovascular disease (11.7%), stroke, and cancer (5.3% for each). The prevalence of limitation to perform BADL was 16.2%. More details on the characteristics of participants can be seen in Table 1.

**Table 1:** Sample description of the 9,389 participants aged 50 and older. Brazilian Longitudinal Study of Aging (ELSI-Brazil), 2015-2016.

Characteristic	%	95%CI
Female Gender	54.0	51.0–57.0
Age group (years)		
50–59	47.7	43.6–51.8
60–69	29.7	27.9–31.5
70–79	15.6	13.8–17.6
80 or older	7.0	5.9–8.4
Urban place of residence	84.7	79.4–88.8
Residence in the regions		
Southeast	47.2	35.6–59.1
South	16.5	8.7–29.0
Midwest	6.6	3.0–13.8
North	5.5	2.3–12.8
Northeast	24.1	15.9–34.9
Education (years)		
None	13.3	11.0–16.0
1 to 4	38.2	36.0–40.5
5 to 8	21.5	19.3–23.8
9 or more	27.0	24.7–29.4
Asset score^a^		
1st quartile	25.1	20.8–30.0
2nd quartile	24.9	23.1–26.7
3rd quartile	25.0	22.7–27.5
4th quartile	25.0	21.8–28.5
Domestic arrangement		
Lives alone	9.0	8.1–10.0
Lives with 1 person	32.2	30.1–34.2
Lives with 2 or more people	58.8	56.3–61.4
Has a private health plan	24.7	22.1–27.4
Hospitalization in the last 12 months (at least one)	10.2	9.3–11.1
Limitation to basic activities of daily living (BADL)^b^	16.2	14.9–17.6
History of medical diagnosis of:		
High blood pressure	52.2	50.3–54.2
Diabetes	15.8	14.6–17.1
Stroke	5.3	4.7–6.0
Cardiovascular disease^c^	11.7	10.6–12.9
Depression	18.6	16.8–20.5
Cancer	5.3	4.7–6.0

All results are expressed as percentages, except when specified. The percentages were estimated considering the sample parameters and the weights of the individuals in the sample.

^a^ Based on the existing equipment in the households and the hiring of domestic workers.

^b^ Difficulty to perform, alone, at least one of the following activities: walking across a room , getting in and out of bed, dressing, bathing, using the toilet and eating.

^c^ Cardiovascular disease: angina, heart failure or myocardial infarction.

The results of the unadjusted analysis of the association between predisposing, enabling and need characteristics and hospital use in the last 12 months are shown in Table 2. Among predisposing and enabling factors, only age, region of residence and education presented statistically significant associations (p < 0.05). All need factors showed a statistically significant association with the outcome.

**Table 2:** Unadjusted analysis of the association between predisposing, enabling and need factors with the occurrence of one or more hospitalizations in the last 12 months among 9,389 participants. Brazilian Longitudinal Study of Aging (ELSI-Brazil), 2015-2016.

Characteristic	Hospitalization in the last 12 months		

	Yes (%)	95%CI	p^d^
Predisposing factors			

Gender			
Female	9.9	8.9–11.0	0.404
Male	10.7	9.2–12.3	
Age group (years)			
50–59	8.7	7.6–10.0	< 0.001
60–69	11.2	9.8–12.8	
70–79	11.6	9.5–14.0	
80 or older	13.6	11.1–16.5	

Enabling factors			

Place of residence			
Urban area	10.1	9.3–10.9	0.455
Rural area	11.2	8.4–14.8	
Residence in the regions			
Southeast	9.9	8.9–11.0	0.044
South	12.1	9.0–15.9	
Midwest	12.4	10.6–14.4	
North	12.7	10.1–15.8	
Northeast	8.6	7.3–10.0	
Education (years)			
None	13.0	11.2–15.1	0.040
1 to 4	9.5	8.1–11.1	
5 to 8	10.5	9.0–12.2	
9 or more	9.6	8.2–11.3	
Asset score^a^			
1st quartile	9.8	8.4–11.4	0.287
2nd quartile	11.2	9.4–13.4	
3rd quartile	10.6	9.2–12.1	
4th quartile	9.3	7.9–10.8	
Domestic arrangement			
Lives alone	10.1	8.5–11.8	0.307
Lives with 1 person	10.9	9.6–12.3	
Lives with 2 or more people	9.9	8.9–11.0	
Healthcare plan			
Yes	11.5	10.1–13.2	0.061
No	9.8	8.8–10.9	

Need factors			

Limitation to basic activities of daily living (BADL)^b^			
Yes	19.0	17.1–21.2	< 0.001
No	8.5	7.7–9.5	
History of medical diagnosis of:			
Hypertension			
Yes	12.5	11.2–13.8	< 0.001
No	7.8	6.9–8.9	
Diabetes			
Yes	15.9	13.8–18.2	< 0.001
No	9.2	8.2–10.2	
Stroke			
Yes	25.8	21.5–30.6	< 0.001
No	9.4	8.5–10.3	
Cardiovascular disease^c^			
Yes	22.5	19.2–26.3	< 0.001
No	8.6	7.8–9.6	
Depression			
Yes	16.2	14.4–18.2	< 0.001
No	8.8	8.0–9.8	
Cancer			
Yes	20.6	17.3–24.7	< 0.001
No	9.6	8.8–10.6	

The percentages were estimated considering the sample parameters and the weights of the individuals in the sample.

^a^ Based on the existing equipment in the households and the hiring of domestic workers.

^b^ Difficulty to perform, alone, at least one of the following activities: walking across a room , getting in and out of bed, dressing, bathing, using the toilet and eating.

^c^ Cardiovascular disease: angina, heart failure or myocardial infarction.

^d^ P-value: Pearson’s chi-squared test.

Table 3 shows the results of the multivariate analysis between predisposing, enabling and need factors, with at least one hospitalization and its frequency in the last 12 months. The following factors had independent and statistically significant associations with one or more hospitalizations: residence in rural versus urban areas (OR = 1.34, 95%CI 1.02-1.76), residence in the Midwest (OR = 1.30; 95%CI 1.05–1.62) and North (OR = 1.50, 95%CI 1.08–2.08) compared to the Southeast region, limitation to perform BADL (OR = 1.77; 95%CI 1.50–2.10) and previous medical diagnosis of chronic diseases, with OR (95%CI), ranging from 1.27 (1.08-1.49) for hypertension to 2.16 (1.70-2.75) for cardiovascular disease. Independent and statistically significant associations with the number of hospitalizations were observed for the age group of 80 years or older (RR = 0.59, 95%CI 0.36–0.99), lives with two or more people (RR = 1.58, 95%CI 1.04–2.42), and medical diagnosis of depression (RR = 1.35, 95%CI 1.02–1.80).

**Table 3:** Multivariate analysis between predisposing, enabling and need factors with the occurrence of at least one hospitalization and their frequency in the last 12 months among 9,134 participants. Brazilian Longitudinal Study of Aging (ELSI-Brazil), 2015-2016.

Characteristic	Hospitalization (yes or no)		Number of hospitalizations (at least one)	
	
	OR	95%CI	RR	95%CI
Predisposing factors				

Gender (*versus* female)				
Male	1.21	0.99–1.47	1.12	0.76–1.64
Age group (*versus* 50–59)				
60–69	1.13	0.93–1.37	0.99	0.70–1.38
70–79	1.06	0.81–1.39	0.78	0.53–1.16
80 or older	1.10	0.79–1.53	0.59^d^	0.36–0.99

Enabling factors				

Residence (*versus* urban area)				
Rural area	1.34^d^	1.02–1.76	1.19	0.85–1.67
Regions (*versus* Southeast)				
South	1.14	0.80–1.63	1.10	0.77–1.59
Midwest	1.30^d^	1.05–1.62	1.10	0.75–1.62
North	1.50^d^	1.08–2.08	0.74	0.40–1.37
Northeast	0.84	0.67–1.06	1.22	0.81–1.85
Education (*versus* 9 years or more)				
None	1.15	0.85–1.54	1.14	0.60–2.16
1 to 4	0.79	0.59–1.06	1.11	0.67–1.84
5 to 8	1.04	0.82–1.31	0.72	0.44–1.18
Asset score^a^ (*versus* 1st quartile)				
2nd quartile	1.08	0.79–1.46	0.71	0.48–1.04
3rd quartile	1.07	0.82–1.41	0.77	0.47–1.28
4th quartile	0.90	0.67–1.21	0.70	0.33–1.47
Domestic arrangements (versus lives alone)				
Lives with 1 person	1.09	0.88–1.34	1.00	0.62–1.62
Lives with 2 or more people	1.11	0.88–1.39	1.58^d^	1.04–2.42
Healthcare plan (*versus* yes)				
No	0.79	0.62–1.02	0.89	0.48–1.66

Need factors				

Limitation to basic activities of daily living (BADL)^b^	1.77^d^	1.50–2.10	1.10	0.85–1.44
History of medical diagnosis of:				
High blood pressure	1.27^d^	1.08–1.49	1.23	0.94–1.61
Diabetes	1.51^d^	1.25–1.83	1.11	0.82–1.51
Stroke	2.10^d^	1.49–2.97	1.03	0.70–1.50
Cardiovascular disease^c^	2.16^d^	1.70–2.75	1.14	0.85–1.52
Depression	1.66^d^	1.39–1.97	1.35^d^	1.02–1.80
Cancer	2.10^d^	1.63–2.70	1.22	0.80–1.85

The percentages were estimated considering the sample parameters and the weights of the individuals in the sample.

Hurdle regression model: odds ratios calculated from logistic regression and relative risks from the Poisson regression and adjusted simultaneously for all variables listed in the table.

^a^ Based on the existing equipment in the households and the hiring of domestic workers.

^b^ Difficulty to perform, alone, at least one of the following activities: walking across a room , getting in and out of bed, dressing, bathing, using the toilet and eating.

^c^ Cardiovascular disease: angina, heart failure or myocardial infarction.

^d^ p < 0.05

The Figure shows estimates of population attributable risks (PAR) associated with the occurrence of at least one hospitalization for different need factors. The results showed higher PAR for medical diagnosis of stroke (PAR = 10.7%, 95%CI 5.0–16.3) and cardiovascular disease (PAR = 10.0%, 95%CI 6.4–13.5 ), followed by cancer (PAR = 8.9%, 95%CI, 5.2–12.7), the limitation to perform BADL (PAR = 6.8%, 95%CI 4.6–8.9), depression (PAR = 5.5%, 95%CI 3.5–7.5), diabetes (PAR = 4.4%, 95%CI 2.2–6.5), and hypertension (PAR = 2.2%; 95%CI 0.8–3.7).

**Figure:**
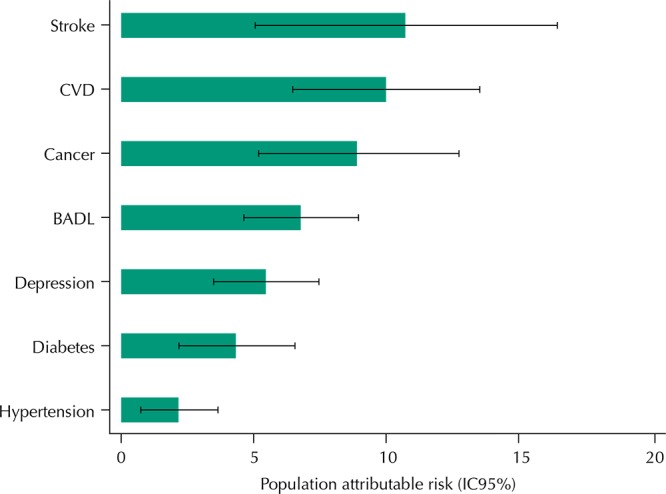
Population attributable risk related to different need factors for hospital use in previous 12 months among 9,134 participants. Brazilian Longitudinal Study of Aging (ELSI-Brazil), 2015-2016.

CVD: cardiovascular disease: angina, heart failure or myocardial infarction; BADL: difficulty performing, alone, at least one of the following activities: walking across a room, getting in and out of bed, dressing, bathing, using the toilet and eating.

## DISCUSSION

The results of this analysis show the importance of need factors for hospital use among older adults. These factors showed stronger associations with the outcome compared to the predisposing and enabling factors of health services usage. Need factors, with one exception (depression), did not reveal statistically significant associations with the number of hospitalizations. Depression was the only need factor associated with both hospital use and their frequency.

The proportion of hospital use may vary among populations, particularly as a function of intrinsic factors (burden of diseases, for example) and the characteristics of health systems. In the ELSI-Brazil population, aged 50 years and older, the proportion of hospital use was 10.2%, reaching 13.6% in the age group of 80 years or older. The proportions of hospitalizations observed in the present analysis were higher than those reported for Mexico in the corresponding age groups (6.4% for those aged 50-59 years and 9.7% in the age group of 70 years or older)^11^, and much lower than those observed among Swedes aged 85 years or older (25%)^9^. In relation to Brazilian studies, the proportion of hospitalizations in the age group of 60 years or older observed in this analysis (11.6%) was similar to that observed in the National Household Sample Survey (PNAD) conducted in 1998, 2003 and 2008 (13.6%, 12.7% and 12.3%, respectively)^19^.

The association between gender and hospital use is controversial. In the Bambui Cohort Study of Aging (Minas Gerais, Brazil)^10^, hospitalizations were more frequent among men, whereas in Scotland, China and Hong Kong, they were more frequent among women^20^. In Mexico, hospitalizations were more frequent among women in the age groups of 50-59 and 60-69 years old, with the opposite being observed in the upper age groups^11^. In the ELSI-Brazil population, there was no association between gender and hospital use, in line with a previous Brazilian national study, using PNAD data^21^, and in a study conducted in Sweden^22^. One of the most consistently observed associations with hospital use is age, with higher proportion in the older age groups^10^ ^,^ ^11^ ^,^ ^20^ ^-^ ^22^. The same association was observed in our analyzes, but it lost statistical significance after adjusting for potential confounding variables.

In the present analysis, as opposed to Mexico^11^, hospital use was higher among residents in rural areas. This use was also a higher among residents in the North and Midwest regions, regardless of other relevant factors. These are the less populous regions of the country, with a lower demographic density and a greater number of remote municipalities, according to the national average^23^, which may hamper the organization and logistics of healthcare networks. Our data are insufficient to explain these results, but it is possible that they are due to difficulties in providing effective primary care to the populations of these regions, to the greater availability of beds in hospitals of less complexity, and to greater barriers to access medium- and high-complexity services^24^.

A recent systematic review has shown that, in most countries, hospital use is not associated with socioeconomic conditions of individuals or their families. In those countries where these differences are observed, they tend to be pro-poor^5^. In Brazil, a study with data from PNAD 1998, 2003 and 2008 showed that the use of health services has become more equitable. The hospital use in previous 12 months prior to the surveys tended to be pro-poor throughout the period, but the difference between the income strata decreased in the most recent year^25^. In the ELSI-Brazil population, educational level showed presented an inverse association with hospital use in the unadjusted analysis, but the association lost statistical significance in the multivariate model. Private health plan coverage and the socioeconomic status of the family, assessed by the asset score, were not associated with hospital use in any analyses.

The influence of the family context on the hospital use has been investigated in different settings. Marital status and the support of friends and relatives were associated with of hospital use in studies conducted in Mexico and Sweden^11^ ^,^ ^22^. Another study conducted among Swedish octogenarians, however, did not show a statistically significant association between these factors and the outcome^9^. In the present analysis, living arrangements showed no association with hospital use, but living with two or more people showed a positive association with the number of hospitalizations.

In this analysis, the only factor associated with both the hospitalizations and their frequency was depression. A previous study conducted in a city in the South of Brazil reported a higher prevalence of depression among hospitalized aged ^26^. In the same study, participants who had been hospitalized reported more feelings of loneliness compared to those not hospitalized. The cross-sectional design of the present investigation does not allow us to know if depression preceded or succeeded hospitalization (reverse causality)^27^ ^,^ ^28^. New analyzes are needed, based on longitudinal studies, to establish the temporality of these associations in older Brazilian adults.

As a population-based study, it was possible to estimate in this investigation the population attributable risks of different diseases and limitation to perform BADL for the hospital use. Population attributable risk is a useful measure for public health because it estimates the proportion of avoidable outcomes if the exposure (chronic condition) is eliminated in the population, considering its prevalence and the magnitude of its association with the outcome. The results of this analysis showed a hierarchy of these conditions for the hospital use. Stroke, cardiovascular disease, cancer and the limitation to perform BADL ranks the first, second, third and fourth places, respectively, followed by depression, diabetes and hypertension.

This study has advantages and limitations. The main advantage is the large population base, with national representation of the population aged 50 or older. In contrast, the study has limitations regarding the discussion of temporality, inherent in the cross-sectional design. Just as the information bias resulting from this type of study cannot be ruled out, it is possible that unmeasured variables may have contributed to the existence of residual confusion in the analyzes. Since the outcome variable is based on the memory of hospitalizations in the last 12 months, there is the possibility of misclassification but, because it is a significant event, it is considered that this possibility is unlikely.

Finally, the results of this study allowed, for the first time in Brazil, to build a hierarchical list of the importance of different diseases and limitation to perform BADL for hospital use among older adults. The results corroborate the fact that need factors are the most important determinants for this use^,^ ^11^ ^,^ ^13^ ^,^ ^21^. Predisposing and enabling factors were less important, which could mean an advance in the fairness of the use of hospital services among Brazilian older adults. However, the place of residence (rural or urban and regions) still remains as a possible impediment to this progress. Four of the diseases that contributed the most to hospitalizations (stroke, cardiovascular disease, diabetes and hypertension) are part of the Brazilian list of primary care sensitive hospitalizations, that is, hospitalizations that can be prevented through effective actions at this level of care^29^. These results show that there is a window of opportunity to reduce unnecessary hospitalizations among older Brazilian adults. In this perspective, primary healthcare policies can contribute to the prevention and better clinical management of morbidities and functional limitation, ultimately reducing avoidable hospitalizations^30^.

## Footnotes

a Fundação Oswaldo Cruz. Brazilian Longitudinal Study of Aging. Rio de Janeiro; c2015 [cited 2017 Nov 28]. Available from: http://elsi.cpqrr.fiocruz.br
